# Structural Modeling of a Novel *CAPN5* Mutation that Causes Uveitis and Neovascular Retinal Detachment

**DOI:** 10.1371/journal.pone.0122352

**Published:** 2015-04-09

**Authors:** Alexander G. Bassuk, Steven Yeh, Shu Wu, Daniel F. Martin, Stephen H. Tsang, Lokesh Gakhar, Vinit B. Mahajan

**Affiliations:** 1 Department of Pediatrics, University of Iowa, Iowa City, IA, United States of America; 2 Omics Lab, University of Iowa, Iowa City, IA, United States of America; 3 Department of Ophthalmology, Emory University School of Medicine, Atlanta, GA, United States of America; 4 Cole Eye Institute, Cleveland Clinic, Cleveland, OH, United States of America; 5 Bernard & Shirlee Brown Glaucoma Laboratory and Barbara & Donald Jonas Laboratory of Regenerative Medicine, Departments of Ophthalmology and Pathology and Cell Biology, Columbia University, New York, NY, United States of America; 6 Department of Biochemistry, University of Iowa, Iowa City, IA, United States of America; 7 Protein Crystallography Facility, University of Iowa, Iowa City, IA, United States of America; 8 Department of Ophthalmology and Visual Sciences, University of Iowa, Iowa City, IA, United States of America; Univeristy of Miami, UNITED STATES

## Abstract

*CAPN5* mutations have been linked to autosomal dominant neovascular inflammatory vitreoretinopathy (ADNIV), a blinding autoimmune eye disease. Here, we link a new *CAPN5* mutation to ADNIV and model the three-dimensional structure of the resulting mutant protein. In our study, a kindred with inflammatory vitreoretinopathy was evaluated by clinical eye examinations, DNA sequencing, and protein structural modeling to investigate the disease-causing mutation. Two daughters of an affected mother demonstrated symptoms of stage III ADNIV, with posterior uveitis, cystoid macular edema, intraocular fibrosis, retinal neovascularization, retinal degeneration, and cataract. The women also harbored a novel guanine to thymine (c.750G>T, p.Lys250Asn) missense mutation in exon 6 of *CAPN5*, a gene that encodes a calcium-activated cysteine protease, calpain-5. Modeling based on the structures of all known calpains revealed the mutation falls within a calcium-sensitive flexible gating loop that controls access to the catalytic groove. Three-dimensional modeling placed the new mutation in a region adjacent to two previously identified disease-causing mutations, all three of which likely disrupt hydrogen bonding within the gating loop, yielding a *CAPN5* with altered enzymatic activity. This is the third case of a *CAPN5* mutation leading to inherited uveitis and neovascular vitreoretinopathy, suggesting patients with ADNIV features should be tested for *CAPN5* mutations. Structural modeling of novel variants can be used to support mechanistic consequences of the disease-causing variants.

## Introduction

Mendelian forms of autoimmune disease are rare. In two large, unrelated kindreds we recently identified the first mutation shown to cause nonsyndromic uveitis: coding mutations in *CAPN5* caused an inherited uveitis called autosomal dominant neovascular inflammatory vitreoretinopathy (ADNIV, OMIM #602537) [1]. ADNIV is characterized by progressive, severe intraocular inflammation, photoreceptor degeneration, retinal neovascularization, intraocular fibrosis, and retinal detachment. Otherwise, affected patients have no associated systemic conditions.

*CAPN5* encodes calpain-5, a calcium-activated signaling protease expressed by retinal photoreceptors [1]. ADNIV-associated mutations in calpain-5 reside in a flexible loop gating the active site and are expected to alter enzymatic activity, likely leading to a gain of function [1]. Since the substrate of calpain-5 has not been identified, however, a functional assay for calpain 5 is not currently available. Nevertheless, for calpains in general, excess activity is associated with retinal and cell degeneration [2–4]. Accordingly, expressing mutant calpain-5 alleles only in the mouse retina is sufficient to trigger disease [5], while deleting the gene (in the knockout mouse) does not result in a phenotype [6]. In this study, we identify a new *CAPN5* mutation in a third ADNIV family and model the functional effect of this mutation on the protein structure.

## Methods

Subjects provided written informed consent under a study approved by the University of Iowa’s Institutional Review Board, and the study adheres to the tenets set forth in the Declaration of Helsinki. Subjects underwent eye exams that included slit-lamp examination, dilated retinal biomicroscopy and indirect ophthalmoscopy, optical coherence tomography, and in selected cases, fluorescein angiography (FFA) and electroretinography (ERG).

### DNA Sequencing and Analysis

Saliva was collected using Oragene sample collection kits (DNAgenotek, Ontario, Canada), and DNA was extracted by salt precipitation. PCR primers were designed as previously described [1], and products were analyzed with a 3730 DNA sequencer and GeneMapper software (Applied Biosystems).

### Structural Analysis

Primary and secondary structure protein alignments and trees were created with Geneious Pro 5.4.6 (http://www.geneiouspro.com). A BLAST search for the catalytic core of calpain-5 against the Protein Database (PDB) returned the structures of the catalytic cores of human calpain-9 with (PDB ID 2P0R; formed catalytic site) and without leupeptin bound (1ZIV; unformed catalytic site) leupeptin bound as the top hits. The sequence identity between target and template sequences was 42%. Other close matches are the catalytic cores of rat calpain-1 with (1TL9) and without leupeptin (1QXP), human calpain-1 (2ARY), human calpain-2 (1KFX), rat calpain-2 (1DF0) and human calpain-8 (2NQA). Homology models were generated using MODELLER 9.14 with the human calpain-9 catalytic cores [7]. The align2d.py python script, which is part of the MODELLER package, was used to generate an alignment between the human calpain-5 and calpain-9 catalytic cores. The model-single.py python script from the MODELLER package was then used to generate 10 models, each using the human calpain-9 catalytic cores (2P0R —apo and 1ZIV—leupeptin bound) as templates. The ten calpain-5 models within each group superimposed well with most variations in loops far away from the ADNIV mutation site. The rmsd over about 260 C_α_ atoms ranged between 0.128–0.232 for the apo template models and between 0.078–0.132 for the leupeptin-bound template models. While the apo and leupeptin-bound template models differed significantly (rmsd of ~ 6.6), this was because of the domain movements on leupeptin binding. The individual domains IIa and IIb align individually quite well with rmsds of 0.435 and 0.566 respectively. Since there was very little variation in the region around our ADNIV mutation site of interest, we picked the top model generated by MODELLER based off the apo-template as a representative model for figures. PyMOL was used to generate all structure figures [8].

## Results

### Phenotypic Ascertainment

**Fig 1:**
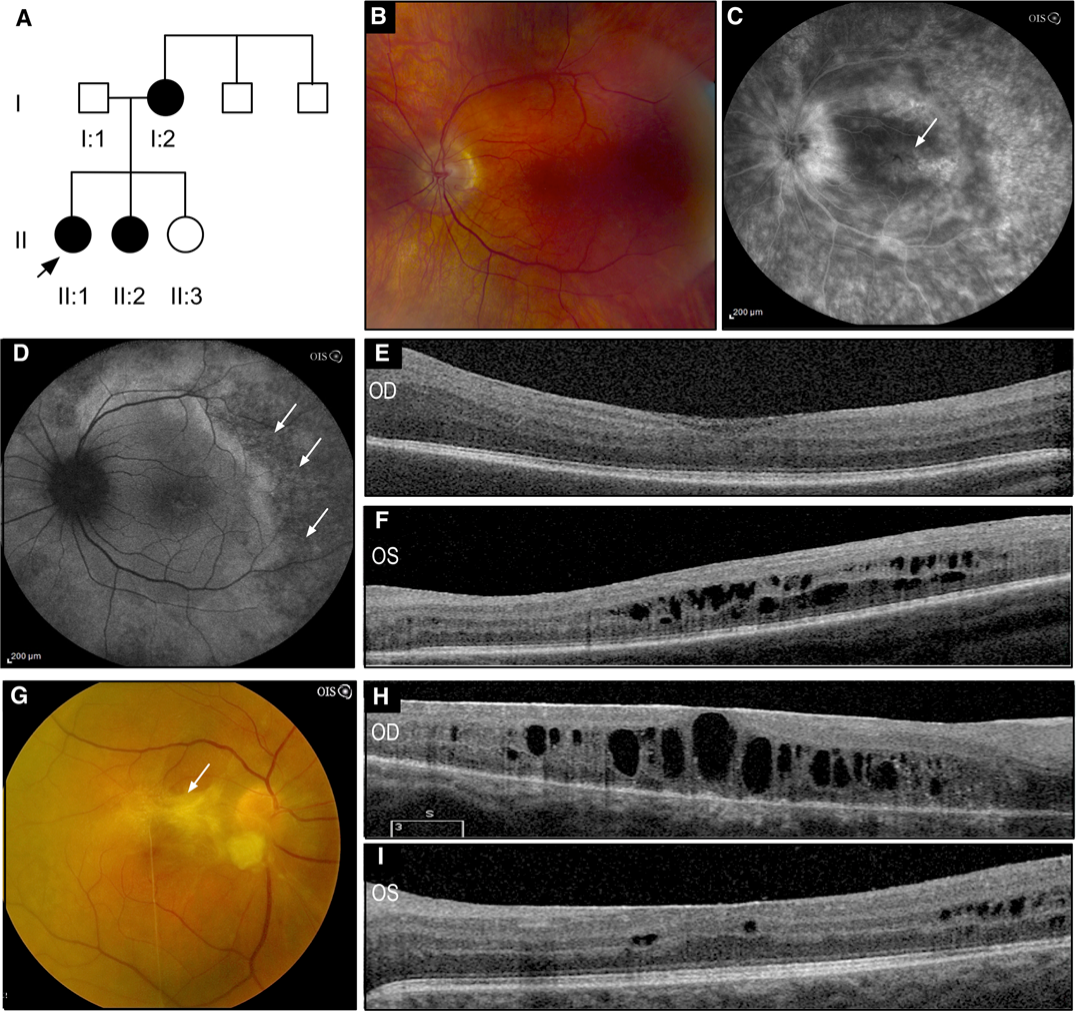
Clinical Imaging. **A**. The pedigree of a family with clinical features of ADNIV did not show a specific pattern of inheritance. Black symbols represent clinically affected subjects. Open symbols represent unaffected subjects. **B**. A color fundus photo of the proband’s (II:1) left macula. **C**. A fluorescein angiogram reveals CME (arrow) and temporal window defects. **D**. Fundus autofluorescence showed a central hyperreflective ring in the macula and hypofluorescence temporal to the macula (arrows). **E, F**. Optical coherence tomography (OCT) shows CME greater in the left eye. **G**. A color fundus photo of the sister’s (II:2) right macula shows a dense epiretinal membrane (ERM, arrow) over the nerve and macula. **H, I**. OCT shows an ERM and CME OU. (ADNIV, autosomal dominant neovascular inflammatory vitreoretinopathy; CME, cystoid macular edema; OCT, optical coherence tomography; OD, right eye; OS left eye; OU, both eyes.)

The proband (II:1) was a 26 year-old female who began to lose vision at age 23. She was diagnosed with noninfectious uveitis with recurrent cystoid macular edema (CME) OU. At the time of her initial presentation, best-corrected visual acuity measured 20/40 OD and 20/200 OS. There was no relative afferent pupillary defect, and intraocular pressures were 12 mmHg OU. Anterior segment examination revealed quiet anterior chambers, 2+ nuclear sclerotic cataracts OU and 1+ posterior subcapsular cataract OS. Dilated funduscopic exam showed 2+ vitreous cell OU, 1+ vitreous haze OS with cystoid macular edema OU (Fig 1), but no bone-spicule pigmentation or pigment clumping, and the periphery was well vascularized. Despite periocular corticosteroid injections and triamcinolone acetonide injections, there was recurrent CME and vitreous inflammation. Over the next six years, she underwent several surgeries in both eyes. These included fluocinolone acetonide (FA) implantation OU for her uveitis and CME, phacoemulsification and intraocular lens implantation for cataracts, Ahmed tube shunts for steroid-response glaucoma, and vitrectomies for vitreomacular traction and epiretinal membranes. She maintained 20/50 OD and 20/100 OS vision for five years until she developed a dense non-clearing vitreous hemorrhage with a tractional detachment associated with peripheral neovascularization OD. This was repaired with vitrectomy, and because of recurrent vitreous hemorrhage in the context of recurrent inflammation, she underwent repeat vitrectomy and repeat FA implantation OU. One year later, her visual acuity measured 20/80 OD and 20/50 OS. She now had diffuse pigmentary spots throughout the peripheral retina. A full-field ERG showed non-recordable cone and rod responses indicative of severe photoreceptor dysfunction OU.

The proband’s sister (II:2) began to lose vision at age 20, also due to noninfectious uveitis. She had undergone surgery for vitreomacular traction and epiretinal membranes OU and cataracts. Her best-corrected visual acuity measured 20/50 OD and 20/30 OS. There was no relative afferent pupillary defect, and intraocular pressures were 16 mmHg OD and 18 mmHg OS. Anterior segment examination revealed subepithelial corneal haze, trace anterior chamber cells, and posterior chamber intraocular lenses OU. Dilated funduscopic examination showed 1+ vitreous cells and a recurrent epiretinal membrane with cystoid macular edema OD (Fig 1). The patient’s VA was maintained at 20/50 OD and 20/40 OS for five years until a decline to 20/150 OD. She developed a dense fibrotic membrane over the macula and a shallow, inferior, 5 clock-hour macula-off rhegmatogenous and tractional retinal detachment. The patient underwent a scleral buckle with cryotherapy OD. Minimal subretinal fluid was present on postoperative day 1. However, on postoperative day 6, she developed an inferior exudative retinal detachment and her vision was reduced to hand motions. Oral prednisone at 60 mg/day was initiated and tapered over six weeks; the exudative RD resolved and an improvement in her visual acuity to 20/125 at postoperative month three. Over the next two years, she underwent two vitrectomies and intravitreal dexamethasone implants for recurrent epiretinal membranes and CME. Nevertheless, her visual acuity declined to 20/400 and the CME recurred. Here left eye showed a similar decline.

The sisters’ mother was reported to have similar vision loss resulting in blindness. Their father and two uncles were not affected (Fig 1). The combination of relentless posterior uveitis, recurrent CME and epiretinal membranes, peripheral pigmentary degeneration, tractional retinal detachment and retinal neovascularization was consistent with Stage III ADNIV in two large pedigrees we previously described. There was no relation to either of these families. The inheritance pattern was compatible with a highly penetrant, dominant Mendelian disease.

### Mutation analysis

**Fig 2:**
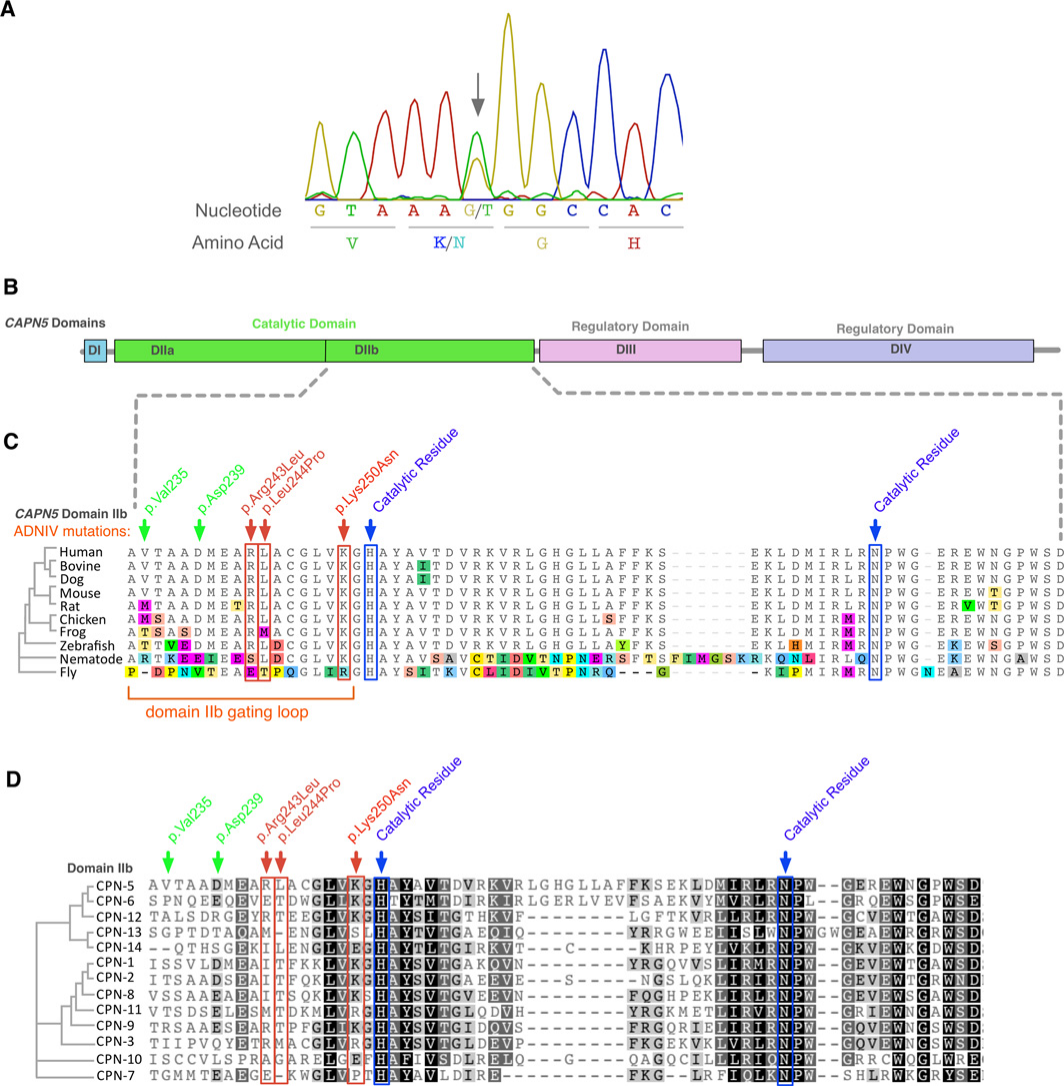
The *CAPN5* gene harbors a novel mutation in exon-6. **A**. Chromatogram of proband showed a heterozygous c.750G>T DNA sequence mutation (arrow) in exon 6 of *CAPN5*. **B**. Calpain-5 is composed of four domains, and the mutation was located in catalytic domain IIb (green). **C**. Primary protein sequence alignment of calpain-5 orthologs shows high evolutionary conservation of the new mutated p.Lys250Asn residue, along with the two prior ADNIV mutations, p.Arg243Leu and p.Leu244Pro (red arrow/box) in catalytic domain IIb. The two previously identified ADNIV mutations and the newly identified ADNIV mutation are 2 to 9 amino acids upstream of the histidine catalytic residue (blue box). Amino acid mismatches are color-highlighted. **D**. Twelve human calpain family paralogs show the p.Lys250Asn is highly conserved. (Black, 100% similarity; dark grey, 80 to 100% similarity; light grey, 60 to 80% similarity; white, less than 60% similarity).

Because of the phenotypic similarity to other ADNIV cases, the *CAPN5* coding region of the family members was sequenced. A heterozygous guanine to thymine nucleotide variant, resulting in a lysine to asparagine substitution (at genomic position Chr11:77115445, NM_004055.4:c.750G>T, p.Lys250Asn), was found in both affected subjects but none of the unaffected (Fig 2). This variant was not present in the dbSNP or 1000 Genome databases. In addition, the variant allele was not found in the over 13,000 *CAPN5* alleles sequenced in the NHLBI Exome Sequencing Project [9].

### Structural Analysis of Calpain-5 ADNIV Mutations

Calpain-5 is a calcium-activated cysteine protease with evolutionarily conserved residues and domains required for protease activity. The calpain-5 catalytic domain-II is encoded by exon-6, where we previously identified two disease-causing *CAPN5* mutations. The newly identified c.750G>T mutation was also found in exon-6, and mutates Lys250, which is within six amino acids of the known mutations and one amino acid away from the catalytic histidine residue. Lys250 is conserved in nearly 100% of vertebrate orthologs. Among the 13 human paralogs, Lys250 is conserved in seven, and in two other calpains the protein has an identically charged arginine (Fig 2).

**Fig 3:**
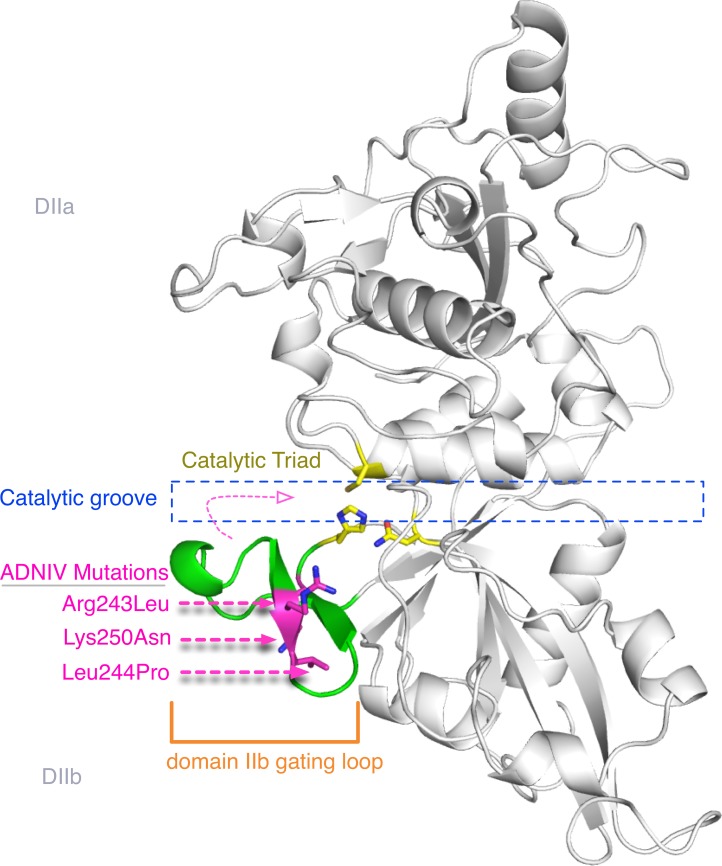
Protein structure modeling of calpain-5 and ADNIV mutants. A three dimensional model of the calpain-5 catalytic domain II was generated using calpain-9 (the closest sequence match) as a template. The residues of the catalytic triad (yellow sticks) are located in the catalytic groove (outlined in blue) between domains IIa and IIb. Although the primary sequence (Fig 2) showed the ADNIV Lys250Asn mutation was several residues away from the two known ADNIV mutations, the three-dimensional model reveals all three ADNIV mutations (magenta sticks) are adjacent to one another on a flexible gating loop (green) that regulates enzymatic activity by controlling access to the catalytic groove. This loop samples many conformations, including a short beta sheet harboring the ADNIV residues as shown here. The mutations alter charged or hydrophobic residues.

The targets of calpain-5 have not been identified and there is no established method for its purification and activity measurement. Since there is no functional assay for calpain-5, we examined how the new ADNIV p.Lys250Asn mutation is structurally related to functional residues in the catalytic domain-II. Homology modeling to calpain-9 generated a three-dimensional structure for the calpain-5 catalytic core (Fig 3) [10, 11]. The two previously identified calpain-5 mutations (p.Arg243Leu, p.Leu244Pro) were modeled as part of a short beta sheet in a flexible gating loop that undergoes calcium-induced conformation changes during enzyme activation (Fig 3). (This location is in contrast to the original homology model that placed these residues in the loop’s alpha helix.) This loop is located on the 5-prime side of the catalytic groove and controls access to substrates. Interestingly, the new calpain-5 mutation sits deeper within the catalytic groove but on the same loop. In the primary sequence, the prior two ADNIV mutations are separated from the newly identified mutation, but when modeled in three-dimensions, the three mutations lie very near one another (Fig 3).

**Fig 4:**
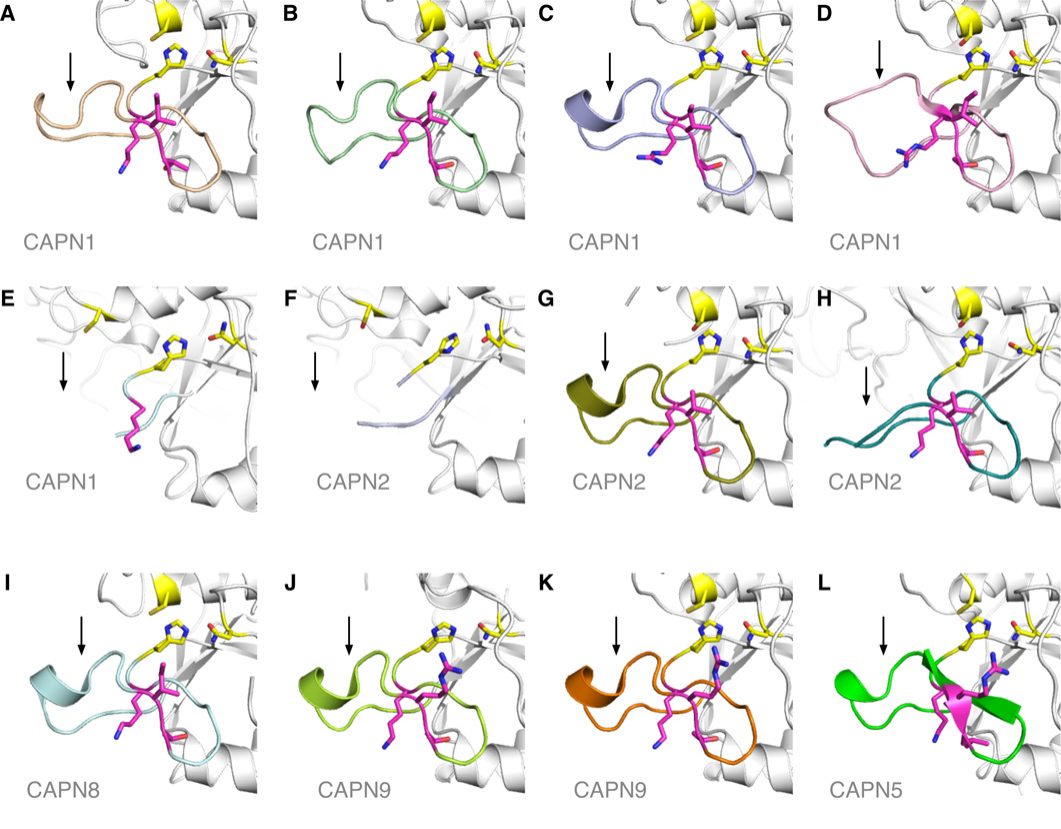
Structure and modeling of the Calpain-5 gating loop. The flexible gating loop (colored differently in each panel) can be observed in different conformations across the known structures of the calpain family. The loops shown here are from the crystal structures of calpain-1 (**A-E**; pdb ids 2ARY, 1ZCM, 1TL9, 1KXR, 1KFX), calpain-2 (**F-H**; 1U5I, 1MDW, 3DF0), calpain-8 (**I**; 2NQA), calpain-9 (**J, K**; 1ZIV, 2P0R) and the homology model of calpain-5 (**L**). The loop in its most ordered form tends to have a short alpha helix (**C, G, I, J, K, L**) and a short beta sheet (**D, L**). Often the loop is disordered and cannot be located in the crystal structure due to lack of sufficient electron density (**E, F, H**). The catalytic residues are shown in yellow and the residues at the site of the calpain-5 ADNIV mutations are shown in magenta.

**Fig 5:**
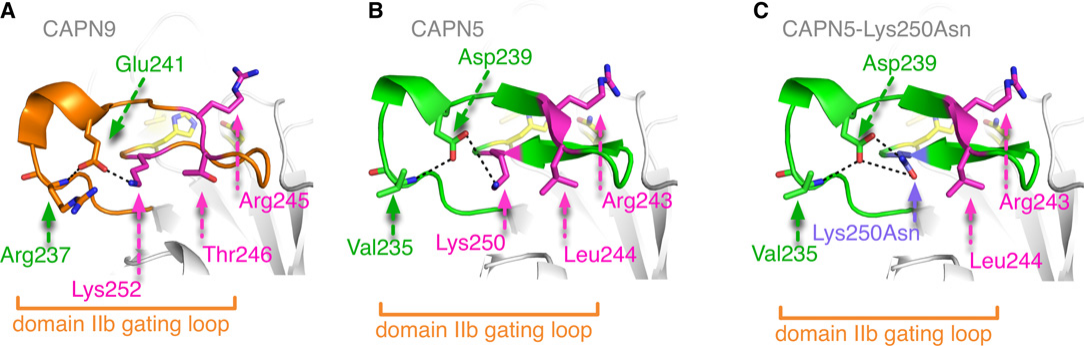
Hydrogen bonding/salt bridge network at the site of p.Lys250Asn ADNIV mutation. **A**. In the crystal structure of calpain-9, Lys252 forms a salt bridge with Glu241, which in turn hydrogen bonds with the backbone nitrogen of Arg237 and locks the flexible gating loop in place. **B**. The same set of interactions are observed in the homology model of calpain-5, where Lys250 is well positioned to form a salt bridge with Asp239, which in turn can form a hydrogen bond with the backbone nitrogen of Val235. **C**. The p.Lys250Asn (purple) mutation modeled here indicates that Asn250, with altered hydrogen bonding capability, will interact differently with Asp239 and affect the conformation and flexibility that this catalytically important gating loop adopts. Changes in the conformation of the gating loop would subsequently alter enzymatic activity.

The gating loop has been found in variable conformations in almost all other calpain crystal structures or left unmodeled due to poor electron density [10, 12–16], indicating that it is highly flexible (Fig 4). Considering the floppy nature of the loop harboring the ADNIV mutations, and the lack of conservation in this stretch [10, 12–16], no single model or template completely captures the different conformations this loop can adopt. However, both the apo and leupeptin bound structures for calpain-9, which best matches the 9-residue stretch spanning the three ADNIV mutations in calpain-5, has this segment of the loop locked in an identical conformation. Therefore, in the context of the catalytic site formation and maintenance, we believe the homology model based on calpain-9 (Fig 5) provides a better perspective on the effect of all the ADNIV mutations identified so far [1]. Since calpains do not have a rigid active site, our structural findings suggest that mutations in this flexible gating loop affect calpain-5 activity. Our identification of a third disease-causing mutation in this same loop supports the idea that the loop provides a mechanism for regulating enzymatic activity [14–16].

The two known ADNIV mutations, p.Arg243Leu and p.Leu244Pro, are at highly conserved residues of calpain-5 orthologs and significantly alter the structure of the gating loop by introducing a hydrophobic leucine in place of a positively charged arginine (p.Arg243Leu) or replacing a hydrophobic residue with a rigid proline (p.Leu244Pro). These mutations likely affect the formation of the alpha helix and beta sheet within this gating loop also observed in other calpain crystal structures and the homology model of calpain-5 (Fig 4) [1].

Our newly identified p.Lys250Asn mutation replaces a longer, positively charged residue with a shorter, polar residue. This modifies an essential hydrogen bond that appears to be necessary for active-site formation and catalysis. Interestingly, eight of the nine calpain-5 paralogs with a conserved positively charged residue at the Lys250 position (Lys252 for calpain-9; Fig 2) also have a conserved negatively charged residue (Asp239 for calpain-5, Glu241 for calpain-9) on a short alpha-helix with which it forms a salt bridge (Fig 5). This Asp/Glu in turn hydrogen bonds with the backbone nitrogen of Val235 (Arg237 for calpain-9; Fig 5), suggesting a hydrogen bond network locks the loop in place. The hydrogen bonding capability of the shorter, polar mutant asparagine is quite different from the longer, positively charged lysine it replaces (Fig 5). The asparagine has the potential to form two hydrogen bonds instead of the single one that lysine can form; this should affect the interaction with Asp239. Taken together, these results suggest that amino acid variants in the region adjacent to the catalytic histidine are not likely to be deleterious, but instead affect the flexible gating loop and catalytic groove, thereby modifying enzymatic activity.

## Discussion

A new, non-synonymous heterozygous mutation in *CAPN5* was identified in clinically affected members of a third family with ADNIV. The clinical phenotype in these patients was similar to the two kindreds reported previously. This new ADNIV family responded to FA implantation in the same way as our other ADNIV patients [17, 18]: FA therapy controlled inflammation and neovascularization, but there was recurrent fibrosis, tractional detachment, and retinal degeneration. Similar to the patients in this report, we have observed asymmetric disease progression between eyes of ADNIV twins [19]. Our combined studies on ADNIV and *CAPN5* mutations suggest that all patients with an ADNIV phenotype should undergo genetic testing for *CAPN5* mutations.

Genes that cause monogenic disorders also harbor polymorphisms that are associated with more common and sometimes less severe disease. It will be important to determine whether any *CAPN5* polymorphisms are risk factors for patients with the ADNIV-like diseases, such as uveitis, diabetic retinopathy, retinitis pigmentosa, or proliferative vitreoretinopathy. Genetic association studies, for example, have identified *CAPN5* variants that are risk factors for type II diabetes, hypertension, high cholesterol, and polycystic ovary syndrome [2, 20–22].

Calpains require careful regulation since they target multiple intracellular proteins and pathways [23–26]. An unregulated calpain-5 could simultaneously activate different intracellular pathways. In comparison to caspases that activate apoptotic, noninflammatory cell death pathways, calpains activate necrotic, inflammatory cell death pathways [27]. This could explain why *CAPN5* retinal degeneration displays a uveitic vitreoretinopathy instead of a noninflammatory photoreceptor degeneration typical of retinitis pigmentosa. The finding that FA therapy treats some calpain-5 effects supports the concept that calpain-5 activates multiple downstream pathways. Nevertheless, the target protein substrates for many calpains, including calpain-5, have yet to be identified [28]. Unless *CAPN5* is directly targeted [29], combination therapies will be required to address the other pathways that trigger photoreceptor degeneration and intraocular fibrosis.

When visualized in the context of the high-resolution, three-dimensional atomic structures, seemingly disparate single mutations, which lie separately along the primary protein sequence, can often be put into perspective. Modeling may be especially illuminating when there is no bioassay to ascertain the effect of a genetic variant. In the absence of experimentally determined structures, suitable templates from the growing number of structures in the PDB can yield homology models that are a good proxy. As we report here, all of the ADNIV mutations identified so far fall in a flexible gating loop and cluster next to the catalytic site; they seemingly affect the formation or movement of this loop during ligand binding or catalysis. In three dimensions, these three mutations cluster together and are part of a backbone or side-chain-mediated hydrogen bond network. The model strongly supports the notion that structure of this peptide loop controls calpain-5 enzymatic activity or specificity. The flexible loops are the most variable region among calpains, and are thought to account for enzymatic differences among calpain isoforms [15, 16, 23]. Minor amino acid substitutions could result in subtle alterations in specificity and catalytic activity differences between isoforms, as well as in disease-causing mutations. It would be worthwhile to genetically screen for variants in *CAPN5* and model them onto a three-dimensional calpain-5 structure to study the commonality of disease-causing mechanisms.

Clinical sequencing is producing a wealth of data, but the functional consequences of the results can often be challenging to interpret. Structural modeling offers insight into underlying pathobiology but, by itself, modeling has limitations. The most powerful genotype-phenotype insights can be made in cases such as the present study, when rigorous clinical phenotype observations coincide with structural analyses.

## References

[1] Mahajan VB, Skeie JM, Bassuk AG, Fingert JH, Braun TA, Daggett HT, et al Calpain-5 mutations cause autoimmune uveitis, retinal neovascularization, and photoreceptor degeneration. PLoS Genet. 2012; 8: e1003001 10.1371/journal.pgen.1003001 23055945 PMC3464205

[2] Huang Y, Wang KK. The calpain family and human disease. Trends Mol Med. 2001; 7: 355–362. 11516996 10.1016/s1471-4914(01)02049-4

[3] Azuma M, Shearer TR. The role of calcium-activated protease calpain in experimental retinal pathology. Surv Ophthalmol. 2008; 53: 150–163. 10.1016/j.survophthal.2007.12.006 18348880 PMC2426920

[4] Vanderklish PW, Bahr BA. The pathogenic activation of calpain: a marker and mediator of cellular toxicity and disease states. Int J Exp Pathol. 2000; 81: 323–339. 11168679 10.1111/j.1365-2613.2000.00169.x PMC2517738

[5] Wert KJ, Skeie JM, Bassuk AG, Olivier AK, Tsang SH, Mahajan VB. Functional validation of a human CAPN5 exome variant by lentiviral transduction into mouse retina. Hum Mol Genet. 2014; 23: 2665–2677. 10.1093/hmg/ddt661 24381307 PMC3990166

[6] Franz T, Winckler L, Boehm T, Dear TN. Capn5 is expressed in a subset of T cells and is dispensable for development. Mol Cell Biol. 2004; 24: 1649–1654. 14749380 10.1128/MCB.24.4.1649-1654.2004 PMC344194

[7] Eswar N, Marti-Renom MA, Webb B, Madhusudhan MS, Eramian D, Shen M-y, et al Comparative Protein Structure Modeling with Modeller Current Protocols in Bioinformatics. Suppl. 15 Hoboken, NJ: John Wiley & Sons, Inc.; 2006 p. 5.6.1–5.6.30. 10.1002/0471250953.bi0506s15 PMC4186674 18428767

[8] PyMOL. New York: Schrödinger; 2014 [cited 11 February 2015]. Available: http://www.pymol.org/.

[9] NHLBI Exome Sequencing Project (ESP). Exome Variant Server. Seattle, WA; 2011 [cited 11 December 2011]. Available: http://evs.gs.washington.edu/EVS/.

[10] Hanna RA, Campbell RL, Davies PL. Calcium-bound structure of calpain and its mechanism of inhibition by calpastatin. Nature. 2008; 456: 409–412. 10.1038/nature07451 19020623

[11] Moldoveanu T, Gehring K, Green DR. Concerted multi-pronged attack by calpastatin to occlude the catalytic cleft of heterodimeric calpains. Nature. 2008; 456: 404–408. 10.1038/nature07353 19020622 PMC2847431

[12] Moldoveanu T, Hosfield CM, Lim D, Elce JS, Jia Z, Davies PL. A Ca(2+) switch aligns the active site of calpain. Cell. 2002; 108: 649–660. 11893336 10.1016/s0092-8674(02)00659-1

[13] Moldoveanu T, Hosfield CM, Lim D, Jia Z, Davies PL. Calpain silencing by a reversible intrinsic mechanism. Nat Struct Biol. 2003; 10: 371–378. 12665854 10.1038/nsb917

[14] Moldoveanu T, Campbell RL, Cuerrier D, Davies PL. Crystal structures of calpain-E64 and-leupeptin inhibitor complexes reveal mobile loops gating the active site. J Mol Biol. 2004; 343: 1313–1326. 15491615 10.1016/j.jmb.2004.09.016

[15] Croall DE, Ersfeld K. The calpains: modular designs and functional diversity. Genome Biol. 2007; 8: 218 17608959 10.1186/gb-2007-8-6-218 PMC2394746

[16] Campbell RL, Davies PL. Structure-function relationships in calpains. Biochem J. 2012; 447: 335–351. 10.1042/BJ20120921 23035980

[17] Tlucek PS, Folk JC, Orien JA, Stone EM, Mahajan VB. Inhibition of neovascularization but not fibrosis with the fluocinolone acetonide implant in autosomal dominant neovascular inflammatory vitreoretinopathy. Arch Ophthalmol. 2012; 130: 1395–1401. 10.1001/archophthalmol.2012.1971 22777573 PMC3885610

[18] Tlucek PS, Folk JC, Sobol WM, Mahajan VB. Surgical management of fibrotic encapsulation of the fluocinolone acetonide implant in CAPN5-associated proliferative vitreoretinopathy. Clin Ophthalmol. 2013; 7: 1093–1098. 10.2147/OPTH.S43939 23785231 PMC3682853

[19] Rowell HA, Bassuk AG, Mahajan VB. Monozygotic twins with CAPN5 autosomal dominant neovascular inflammatory vitreoretinopathy. Clin Ophthalmol. 2012; 6: 2037–2044. 10.2147/OPTH.S40086 23271883 PMC3526908

[20] Baier LJ, Permana PA, Yang X, Pratley RE, Hanson RL, Shen GQ, et al A calpain-10 gene polymorphism is associated with reduced muscle mRNA levels and insulin resistance. J Clin Invest. 2000; 106: R69–73. 11018080 10.1172/JCI10665 PMC387246

[21] Horikawa Y, Oda N, Cox NJ, Li X, Orho-Melander M, Hara M, et al Genetic variation in the gene encoding calpain-10 is associated with type 2 diabetes mellitus. Nat Genet. 2000; 26: 163–175. 11017071 10.1038/79876

[22] Sáez ME, Martínez-Larrad MT, Ramírez-Lorca R, González-Sánchez JL, Zabena C, Martinez-Calatrava MJ, et al Calpain-5 gene variants are associated with diastolic blood pressure and cholesterol levels. BMC Med Genet. 2007 10.1186/1471-2350-8-1 PMC1783645 17227582

[23] Croall DE, DeMartino GN. Calcium-activated neutral protease (calpain) system: structure, function, and regulation. Physiol Rev. 1991; 71: 813–847. 2057527 10.1152/physrev.1991.71.3.813

[24] Sato K, Kawashima S. Calpain function in the modulation of signal transduction molecules. Biol Chem. 2001; 382: 743–751. 11517927 10.1515/BC.2001.090

[25] Goll DE, Thompson VF, Li H, Wei W, Cong J. The calpain system. Physiol Rev. 2003; 83: 731–801. 12843408 10.1152/physrev.00029.2002

[26] Zatz M, Starling A. Calpains and disease. N Engl J Med. 2005; 352: 2413–2423. 15944426 10.1056/NEJMra043361

[27] Syntichaki P, Xu K, Driscoll M, Tavernarakis N. Specific aspartyl and calpain proteases are required for neurodegeneration in C. elegans. Nature. 2002; 419: 939–944. 12410314 10.1038/nature01108

[28] DuVerle DA, Ono Y, Sorimachi H, Mamitsuka H. Calpain cleavage prediction using multiple kernel learning. PLoS One. 2011; 6: e19035 10.1371/journal.pone.0019035 21559271 PMC3086883

[29] Nelson NG, Skeie JM, Muradov H, Rowell HA, Seo S, Mahajan VB. CAPN5 gene silencing by short hairpin RNA interference. BMC Res Notes. 2014; 7: 642 10.1186/1756-0500-7-642 25216694 PMC4169796

